# Using Ascorbate Oxidase to Minimize Ascorbate Interference with Glycerol Quantification

**DOI:** 10.3390/mps9050146

**Published:** 2026-10-09

**Authors:** Christina M. N. Cheng, Daniel S. Lark, Christopher Bell

**Affiliations:** Department of Health and Exercise Science, Colorado State University, Fort Collins, CO 80523-1582, USA; c.cheng@colostate.edu (C.M.N.C.); dan.lark@colostate.edu (D.S.L.)

**Keywords:** vitamin C, ascorbic acid, lipolysis, Trinder-based assay, antioxidant

## Abstract

Intravenous delivery of the antioxidant, ascorbate, is a common perturbation in human physiology studies of oxidative stress and metabolic regulation. Lipolysis, an important step in the metabolism of lipids, is typically represented by circulating concentrations of glycerol. Trinder-based assays are often used in clinical research to quantify glycerol concentration, but the presence of ascorbate confounds these assays, thus creating a challenge for studies interested in both oxidative stress and lipolysis. The purpose of the current study was to develop a protocol using ascorbate oxidase (AO) to minimize the interference of ascorbate during the quantification of glycerol concentration in human plasma. A healthy adult, who did not supplement their diet with oral or intravenous ascorbate, provided plasma. Ascorbate was added to each sample resulting in a final concentration of 2.0 mmol/L. 100 µL of AO solution was added to 400 µL ascorbate-spiked samples to produce AO concentrations ranging from 2 to 200 U/mL. The presence/absence of ascorbate was determined in each sample. An AO concentration of 20 U/mL, combined with a 30 min incubation, resulted in undetectable ascorbate in ascorbate-spiked samples. In plasma samples collected from humans receiving intravenous ascorbate, the quantification of glycerol yielded unusually low values. However, pretreatment with AO resulted in ascorbate concentrations below the level of detection and yielded glycerol values that did not differ from control (“ascorbate-free”) plasma. In clinical studies of oxidative stress and lipolysis, interference by ascorbate in Trinder-based quantification of glycerol may be minimized by pre-treatment with 20 U/mL AO and a 30 min incubation.

## 1. Introduction

Intravenous delivery of the antioxidant, ascorbate, is a common perturbation in human physiology studies of oxidative stress [1,2,3]. If decreased oxidative stress improves physiological function, the original dysfunction could be attributed to increased reactive oxygen species production and/or decreased antioxidant defenses.

The dysfunctional metabolism of lipids has enormous clinical relevance in many human chronic diseases. The first step in the utilization of lipids, specifically triglycerides, is lipolysis, the breakdown of triglycerides into free fatty acids and glycerol. While circulating free fatty acids and glycerol both reflect lipolysis, glycerol is widely considered to be the superior marker. Free fatty acids released during lipolysis, instead of undergoing further catabolism (mitochondrial β-oxidation, peroxisomal oxidation, enzymatic pathways (e.g., cyclooxygenase, lipoxygenase)), may be re-esterified back into triglycerides by acyl-CoA synthetase enzymes. In contrast, glycerol cannot be re-esterified and thus more accurately reflects lipolysis [4].

In smaller research facilities lacking expensive high-resolution analytical apparatus (i.e., tandem mass spectroscopy, etc.), circulating glycerol is usually quantified using inexpensive Trinder-based assays [5,6,7]. These assays are colorimetric tests that typically rely on a coupled enzymatic reaction that (1) oxidizes the substrate to yield hydrogen peroxide and (2) catalyzes reaction of hydrogen peroxidase with 4-aminioantipyrine and a phenol to generate a colored quinoneimine dye [8]. Unfortunately, these assays are confounded by ascorbate, which inhibits quinoneimine dye production by reducing hydrogen peroxide prior to dye formation [9], creating a challenge for clinical studies of oxidative stress and lipolysis. Many glycerol kits, (e.g., Cayman Chemical, Ann Arbor, MI, USA), do not explicitly alert users to the risk of antioxidant interference, thus creating potential problems for physiologists and laboratory scientists unfamiliar with the redox mechanism. Validating a method of diminishing ascorbate interference is important for ensuring the accuracy of circulating glycerol quantification in laboratory and clinical settings where ascorbate interference is suspected.

In previous clinical studies of cholesterol and triglyceride regulation, ascorbate oxidase (AO) was proposed as a method of minimizing ascorbate interference in Trinder-based assays [10,11]. AO catalyzes the oxidation of ascorbate to dehydroascorbate, which has no reducing power. AO pre-treatment efficacy for removing ascorbate interference from blood samples has been previously demonstrated for the quantification of cholesterol and triglyceride, but not for the quantification of glycerol. Accordingly, the aim of the current study was to develop a protocol using AO to minimize ascorbate interference with glycerol quantification in human plasma. AO was tested at three concentrations (2, 20, and 200 U/mL) to determine a feasible concentration for use in plasma samples.

## 2. Materials and Methods

The work described herein was undertaken as part of a registered clinical trial (NCT07341308) investigating the potential interaction between oxidative stress and thermogenic/metabolic function in adult humans. The general aim of the trial was to determine the potential influence of oxidative stress on the metabolic responses to pharmacological stimulation. Metabolic responses were compared during the co-administration of saline (the control condition) and the antioxidant, ascorbic acid. The Institutional Review Board at Colorado State University (FWA0000647) approved the study (Protocol #6115), consistent with the Declaration of Helsinki.

### 2.1. Exogenous Ascorbate Spiking Test

To determine if ascorbate could be eliminated from plasma, whole blood was sampled from an antecubital vein of a healthy young adult woman who did not supplement with ascorbate. Blood was collected in a pre-chilled tube coated with ethylenediaminetetraacetic acid (EDTA) and transferred to ice. Within 10 min of collection, the tube was centrifuged (3600× *g*, 4 °C, 10 min), and plasma was aliquoted into three phials and stored for six days at −80 °C.

Previous human ascorbate infusion studies [1,2,3], using infusion protocols similar to ours, suggest plasma ascorbate concentrations in the current study would not exceed 2.0 mmol/L. Accordingly, two human plasma phials were spiked with ascorbate (ASCOR, McGuff Pharmaceuticals, Santa Ana, CA, USA) at a concentration of 2.0 mmol/L; the remaining phial served as a baseline control. Lyophilized AO (MilliporeSigma, St. Louis, MO, USA; LOT #4341624, 380.0 U/mg) was stored at 4 °C prior to reconstitution in 1 mL saline by inversion to create a 1000 U/mL stock and then underwent serial dilution twice to generate 100 U/mL and 10 U/mL solutions. An amount of 100 µL of each AO solution was added to 400 µL of ascorbate-spiked plasma, generating three spiked plasma samples at AO concentrations of 2, 20, and 200 U/mL. Ascorbate concentration was semi-quantified from the AO-spiked plasma samples every 5 min using test strips (Quantofix, Macherey-Nagel, Düren, Germany) with a visual measurement range of 0–2000 mg/L ascorbate and a lower detection limit of 50 mg/L ascorbate.

### 2.2. Clinical Trial Protocol: Stimulation of Lipolysis and Ascorbate Infusion

The clinical trial (NCT07341308) involved two separate intravenous infusions, in young healthy adult humans, performed on separate days, in a pseudo-random order. Eligibility criteria included age between 18 and 40 years, stable body weight (weight change < 5 lbs. in the past 6 months), and body mass index between 25 and 30 kg/m^2^. Participants were excluded if they reported pregnancy, breastfeeding, smoking, recreational drug usage, sulfite allergy, vitamin D supplementation within the last 4 weeks, antioxidant supplementation within the last 4 weeks, or use of medications known to interfere with lipid metabolism or oxidative stress. Participants were also excluded if they self-reported any of the following: autonomic disorder, liver disease, cardiovascular disease, kidney disease, bladder disorder, hypertension, dyspnea, or diabetes. Lipolysis was stimulated using the non-selective beta-adrenergic receptor agonist, isoproterenol, in three 30 min consecutive, continuous doses (6, 12, and 24 ng/kg fat-free mass/min). On one occasion, isoproterenol was co-infused with saline (1 mL/min), and on the other, with ascorbate (ASCOR, McGuff Pharmaceuticals, Santa Ana, CA, USA) diluted in 100 mL saline (0.05 g/kg fat-free mass at 1 mL/min). The exact concentration of ascorbate infused depended on the participant’s fat-free mass. Venous blood was sampled at baseline and after each isoproterenol dose using the methods described in the previous section.

### 2.3. Ascorbate Oxidase Pre-Treatment for Glycerol Quantification

Plasma glycerol was analyzed using a Trinder-based colorimetric glycerol assay (Cayman Chemical, Ann Arbor, MI, USA). Lyophilized AO (MilliporeSigma, St. Louis, MO, USA; LOT #4341624, 380.0 U/mg) was stored at 4 °C prior to reconstitution in 5 mL saline by inversion to create a 200 U/mL stock solution. After loading 50 µL of all standards and samples into a 96-well plate, 5.6 µL AO was pipetted into each well for a final concentration of 20 U/mL. All standards and samples received the same volume of AO, so a dilution correction was not needed. All standards and samples were analyzed in duplicate. Duplicates with a coefficient of variation (CV) > 15% were considered unreliable. The plate was gently mixed at room temperature for 30 min. The remaining steps in the glycerol assay kit were performed according to manufacturer guidelines.

To evaluate if the AO pre-treatment itself affects glycerol measurement, glycerol standard concentrations for standards pre-treated with AO were back-calculated from its fitted calibration curve and compared with the given concentration for each of the two plates that were run. Recovery and bias were then calculated.

### 2.4. Statistical Analysis

Statistical calculations were performed using RStudio 2024.12.0 (Posit Software, Boston, MA, USA). Normality was assessed using histograms, Q-Q plots, and the Shapiro–Wilks test. A linear mixed model was used to analyze plasma glycerol over time and between conditions with the lme4 (version 1.1.37) and lmerTest (version 3.1.3) packages. Two data points exceeded the CV threshold (CV = 20.4% and 25.2%) but did not change the overall results, so they were not excluded. Post hoc tests were conducted using Tukey’s Honestly Significant Difference tests with the emmeans package. Significance was set at α = 0.05.

## 3. Results

### 3.1. Ascorbate Oxidase Renders Ascorbate Undetectable in Ascorbate-Spiked Samples and Plasma Samples from the Ascorbate Clinical Trial

**Figure 1:**
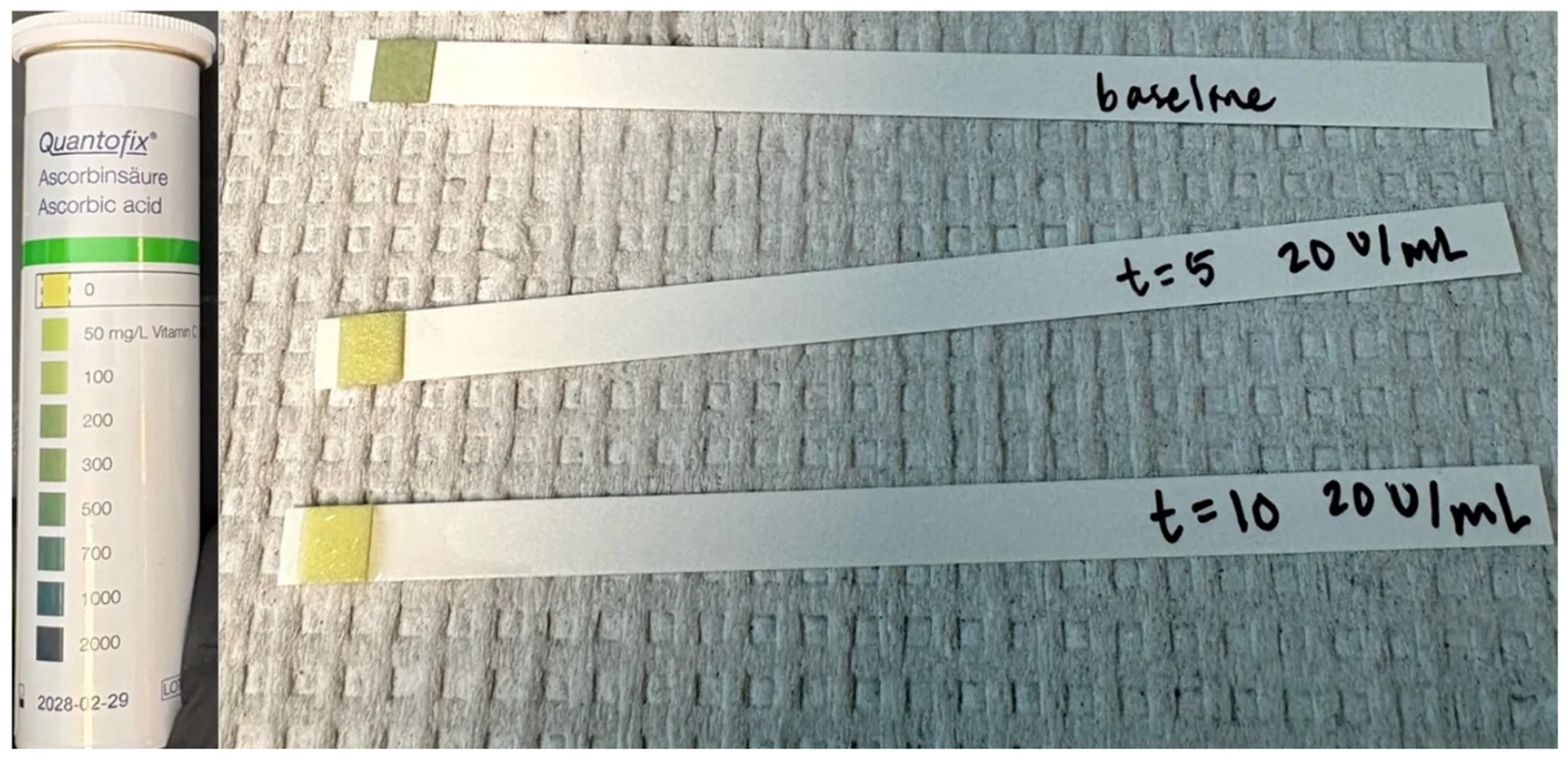
Ascorbate detection using Quantofix test strips in a plasma sample taken after 105 min of continuous ascorbate infusion. Top strip is baseline. Middle strip represents 20 U/mL AO after 5 min incubation. Bottom strip represents 20 U/mL AO after 10 min incubation.

A sample concentration of 20 U/mL AO, coupled with a 30 min incubation, appeared to render ascorbate below the limit of detection (i.e., <50 mg/L) within the ascorbate-spiked plasma samples. We next determined if AO eliminates ascorbate in plasma collected at the end of ascorbate infusion. A total of 10 min of incubation with AO rendered ascorbate visually undetectable (Figure 1).

### 3.2. Ascorbate Oxidase Appears to Minimize Ascorbate Interference with Glycerol Quantification

**Figure 2:**
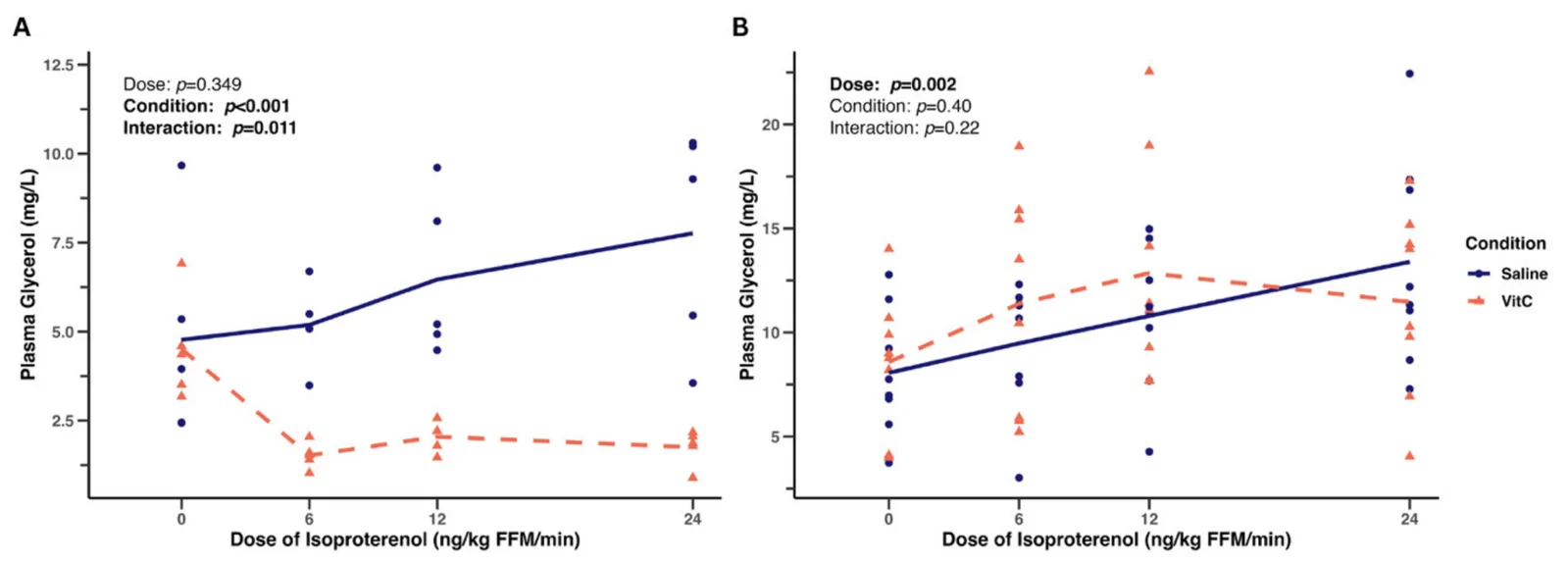
Plasma glycerol response during incremental beta-adrenergic receptor stimulation with and without co-infusion of ascorbate. Lines represent mean values, and symbols represent individual participant responses. (**A**) Plasma glycerol appeared lower with co-infusion of ascorbate when AO pre-treatment was not used (*n* = 5). (**B**) Plasma glycerol was not different between conditions when AO pre-treatment was used (*n* = 8).

Isoproterenol alone raised plasma glycerol by up to 63% (Figure 2A), indicative of increased lipolysis. However, co-infusing ascorbate with isoproterenol decreased glycerol (Figure 2A), generating unusually low concentrations across all timepoints in a portion of the sample (*n* = 5) analyzed part-way through the clinical trial. This could be interpreted as attenuated beta-adrenergic receptor mediated lipolysis, an explanation that could be considered non-physiological. Alternatively, and more realistically, the likely explanation is ascorbate interference with glycerol quantification.

Upon conclusion of the larger trial, plasma glycerol concentrations in the entire sample (n = 8) were quantified and are displayed in Figure 2B. Participants included 5 males and 4 females, aged 26 ± 6 years. When all samples were pretreated with AO, coupled with a 30 min incubation, glycerol concentrations increased with increasing isoproterenol doses in both saline and ascorbate conditions. The concentrations aligned with the values expected during pharmacological-stimulated lipolysis.

### 3.3. Ascorbate Oxidase Effect on Assay Performance

For Plate 1, AO-treated glycerol standards (2–20 µg/mL) had a recovery of 92.8–104.6% and a bias of −7.2–11.3%. The standard curve had strong linearity (R^2^ = 0.991) and precision (CV: 0.07–4.97%) (Table 1). For Plate 2, AO-treated glycerol standards (2–20 µg/mL) had a recovery of 96.8–105.2% and a bias of −3.2–5.2%. The standard curve had strong linearity (R^2^ = 0.997) and precision (CV: 0.04–4.43%) (Table 2).

Between-run reproducibility of AO-treated glycerol standards was assessed by calculating the inter-assay CV, ranging from 2.23 to 8.20%. For reference, the manufacturer’s (i.e., without AO treatment) reported linearity is R^2^ = 0.99, intra-assay CV is 2.4–7.0%, and inter-assay CV is 8.1–11.4%.

## 4. Discussion

Trinder-based assays are susceptible to interfering agents like ascorbate. This study aimed to develop a protocol using AO to minimize ascorbate interference with glycerol quantification in human plasma. High-dose ascorbate infusion (plasma concentration ≤ 2 mmol/L) interfered with Trinder-based glycerol quantification, generating unusually low values. AO pre-treatment (20 U/mL with 30 min incubation) visually appeared to decrease ascorbate interference under the described test conditions. Trinder-based assays are commonly used in clinical and research settings and known to be disrupted by ascorbate due to interaction with the hydrogen peroxide generated to produce the chromophore [9,10,11]. In studies manipulating oxidative stress, unexpectedly low results from a Trinder-based assay should be investigated for potential ascorbate interference.

A primary limitation of this study is the lack of more sensitive independent reference methods for measuring glycerol and ascorbate concentrations. As this study was unfunded and emerged as a troubleshooting project amongst an ongoing clinical trial, we were unable to perform such analyses due to feasibility constraints and acknowledge that using these analytical methods would strengthen the results to be more definitive. The study was also limited by the use of plasma donated by only one research participant for the ascorbate spiking test. While it is true that multiple donors would strengthen the spiking test due to variability in plasma composition, the characteristics of the donor in this study were specific to the characteristics of the participant sample of the larger clinical trial. Additionally, this study was limited by the lack of a dedicated experiment confirming that AO treatment does not affect the glycerol assay chemistry itself. Based on the strong glycerol standard recovery in this study and the use of AO in similar assays without reported interference [10,11], we suggest that it is unlikely that AO interferes with the glycerol assay mechanism. Future methodology studies specifically aimed at validating AO pre-treatment of samples for Trinder-based glycerol quantification should incorporate gold-standard reference methods (e.g., GC-MS, HPLC), multiple plasma donors, and independent validation that AO treatment does not affect the assay mechanism.

Our data suggest that enzymatic treatment with AO (20 U/mL with 30 min incubation) enables colorimetric Trinder-based glycerol quantification in human plasma after ascorbate infusion. Ascorbate is a commonly consumed supplement and is frequently used for its antioxidant properties in research. Future studies should use a reference method to quantitatively validate the AO minimization of ascorbate interference. Clinicians and scientists should be aware of ascorbate interference with glycerol assays and consider AO pre-treatment.

## Figures and Tables

**Table 1:** Back-calculated concentrations for glycerol standards pre-treated with AO for Plate 1. Recovery and bias were calculated by back-calculating glycerol standard concentrations according to the fitted standard curve and comparing to the nominal values.

Nominal Concentration (µg/mL)	Absorbance	Back-Calculated Concentration (µg/mL)	Recovery (%)	Bias (%)
2.0	0.032	2.23	111.3	11.3
4.0	0.067	4.19	104.6	4.6
6.0	0.101	6.09	101.5	1.5
8.0	0.135	7.99	99.9	−0.1
10.0	0.166	9.73	97.3	−2.7
15.0	0.241	13.92	92.8	−7.2
20.0	0.365	20.86	104.3	4.3

**Table 2:** Back-calculated concentrations for glycerol standards pre-treated with AO for Plate 2. Recovery and bias were calculated by back-calculating glycerol standard concentrations according to the fitted standard curve and comparing to the nominal values.

Nominal Concentration (µg/mL)	Absorbance	Back-Calculated Concentration (µg/mL)	Recovery (%)	Bias (%)
2.0	0.057	1.98	99.1	−0.9
4.0	0.113	3.90	97.6	−2.4
6.0	0.170	5.86	97.6	−2.4
8.0	0.225	7.74	96.8	−3.2
10.0	0.306	10.52	105.2	5.2
15.0	0.447	15.36	102.4	2.4
20.0	0.572	19.64	98.2	−1.8

## Data Availability

The original contributions presented in the study are included in the article, further inquiries can be directed to the corresponding author.
